# The anti-arthritic activity of total glycosides from *Pterocephalus hookeri*, a traditional Tibetan herbal medicine

**DOI:** 10.1080/13880209.2016.1263869

**Published:** 2016-12-09

**Authors:** Xiao-fei Shen, Yong Zeng, Jia-chuan Li, Ce Tang, Yi Zhang, Xian-li Meng

**Affiliations:** a College of Pharmacy, Chengdu University of Traditional Chinese Medicine, Chengdu, Sichuan, China;; b College of Ethnic Medicine, Chengdu University of Traditional Chinese Medicine, Chengdu, Sichuan, China

**Keywords:** Tibetan herbal medicine, *Pterocephalus hookeri* (C. B. Clarke) Hock, total glycosides, adjuvant-induced arthritis, anti-arthritic activity, anti-inflammatory and analgesic activities, NF-κB, oxidative stress

## Abstract

**Context:***Pterocephalus hookeri* (C. B. Clarke) Hock., a traditional Tibetan herbal medicine rich in glycosides, has been used to treat several diseases including rheumatoid arthritis.

**Objective:** To evaluate the anti-arthritic activity of total glycosides from *P. hookeri*, and its possible mechanisms of action.

**Materials and methods:** Anti-arthritic activity of total glycosides from *P. hookeri* (oral administration for 30 days at 14–56 mg/kg) was evaluated using paw swelling, arthritis scores and histopathological measurement in adjuvant-induced arthritis (AA) Sprague-Dawley rats. The NF-κB p65 expression in synovial tissues, and serum superoxide dismutase (SOD) activity, malondialdehyde (MDA) and nitric oxide (NO) levels was measured in AA rats, respectively. Further assessment of anti-inflammatory and analgesic activities of these glycosides were carried out using inflammation and hyperalgesia models induced by xylene, carrageenan, agar and acetic acid, respectively.

**Results:** Total glycosides (56 mg/kg) decreased the paw swelling (38.0%, *p* < 0.01), arthritis scores (25.3%, *p* < 0.01) and synovial inflammation in AA rats. The glycosides significantly (*p* < 0.05–0.01) attenuated the inflammation induced by xylene, carrageenan, acetic acid and agar, increased the pain threshold in acetic acid-induced writhing in mice and mechanical stimuli-induced hyperalgia in AA rats. The glycosides (14, 28, 56 mg/kg) also suppressed the NF-κB p65 expression (33.1–78.2%, *p* < 0.05–0.01), reduced MDA (21.3–35.9%, *p* < 0.01) and NO (20.3–32.4%, *p* < 0.05–0.01) levels, respectively, enhanced the SOD activity (7.8%, *p* < 0.05) at 56 mg/kg in AA rats.

**Discussion and conclusion:** Our findings confirmed the anti-arthritic property of the total glycosides from *P. hookeri*, which may be attributed to its inhibition on NF-κB signalling and oxidative stress.

## Introduction

Rheumatoid arthritis (RA), a common autoimmune disease caused by multiple reasons, is characterized by persistent synovitis, infiltration of inflammatory cells, vascular proliferation and progressive cartilage and bone damage (McInnes & Schett 2011). Associated with progressive disability, systemic complications and premature death, this disease affects approximately 1% of the world population, and results in high socioeconomic costs of treatment (Gibofsky 2012). Although the pathogenesis of RA has not yet been fully understood, it has been suggested that the abnormal activation of inflammatory signalling pathways and subsequently excessive oxidative stress play a critical role in the development of RA (Stamp et al. 2012). The understanding that the RA is driven by maladaptive and nonresolving inflammation that leads to the clinical application of anti-inflammatory therapy including using glucocorticoids, nonsteroidal anti-inflammatory drugs (NSAIDs) and disease-modifying agents of rheumatoid diseases (DMARDs) (Singh et al. 2012). Despite the progress made in the treatment of RA, the long-term benefits of these medication in reducing the risk of joint damage and functional decline are still largely unknown (Goekoop-Ruiterman et al. 2005). Furthermore, long-term application of anti-inflammatory agents may result in severe side effects including gastrointestinal toxicity, cardiovascular risk, bone marrow suppression and kidney damage (Bermas 2014). Thus, it is necessary to develop alternative therapeutic strategies with higher effects and safeties yet lower cost to treat RA.

*Pterocephalus hookeri* (C. B. Clarke) Hock., one of the most popular traditional Tibetan herbs, locally known as ‘Bang-Zi-Du-Wu’, is recorded in the Tibetan medical classic named ‘rGyud-bZhi’ (usually known as the Four Tantras) and the 2010 edition of Pharmacopoeia of China. Traditionally, *P. hookeri* has been widely applied in many Tibetan herbal medicine formulations to treat multiple diseases including common cold, RA and enteritis, dysentery, etc. (Pharmacopoeia Committee 2010). Recent studies indicate that the main effective components of *P. hookeri* are glycosides, such as iridoid glycosides (loganin and cantleyoside) and triterpenoid saponins (hookerosides A-D) (Tian et al. 1993; Wu et al. 2014a, 2014b). Furthermore, Zhang et al. (2009) demonstrated that the ethanol and aqueous extracts of *P. hookeri* exhibited obvious anti-inflammatory and analgesic effects with less toxicity. Although *P. hookeri* has been used to treat inflammation-related diseases in traditional Tibetan medicine for a long period, there is little scientific evidence showing its effect on RA. Moreover, previous studies mainly focused on the pharmacological effects of the crude extracts of *P. hookeri* without studying its main active constituents, such as the total glycosides. Herein, the purpose of this study is to evaluate the anti-RA activity of the total glycosides from *P. hookeri* using animal models with the expectation to develop an alternative strategy in the treatment of RA.

## Materials and methods

### Preparation of the total glycosides from *P. hookeri*

**Figure 1.**
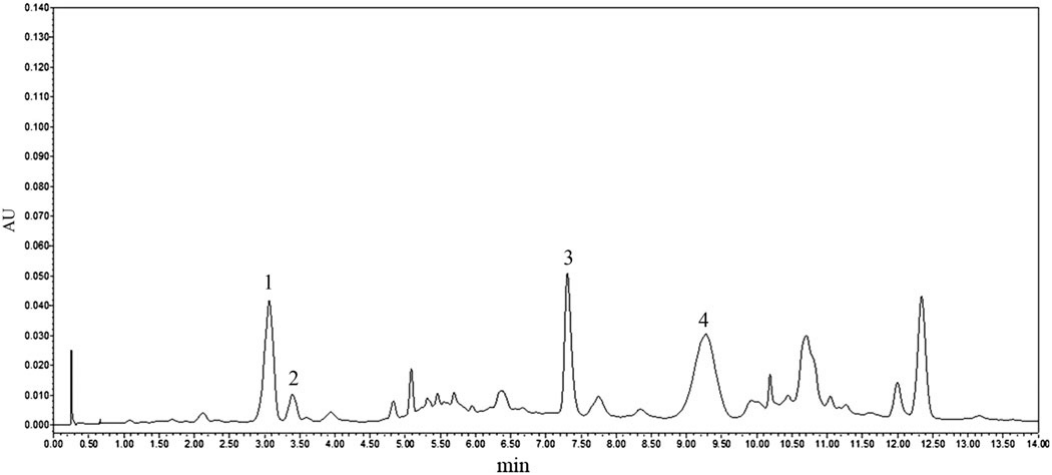
UPLC chromatogram of total glycosides from *P. hookeri*. Four compounds were deduced by comparing individual peak retention times with those of the standard substances. The extracts were analyzed by Waters Acquity UPLC-photodiode array (Waters, Milford, MA, USA). The chromatographic separation was carried out on Acquity UPLCR BEH C18 (2.1 mm ×50 mm, 1.7 μm) with the column temperature at 35 °C. The mobile phase was acetonitrile (A) with 0.2% phosphoric acid solution (B) in a gradient mode which was described as follows: 0–4 min, 10% A and 90% B; 5–10 min, 15% A and 85% B; 11–14 min, 20% A and 80% B. The flow rate was 0.5 mL/min and the PDA UV wavelength was 273 nm. The four main compounds of total glycosides from *P. hookeri* were identified, including sweroside (peak 1), loganin (peak 2), sylvestroside I (peak 3) and cantleyoside (peak 4).

The entire plants of *P. hookeri* were collected from Garzê in Sichuan Province (No. 20100809), and identified by Professor Yi Zhang, College of Ethnic Medicine, Chengdu University of Traditional Chinese Medicine. The plant materials were powdered, and extracted twice with 95% ethanol (1.5 h each time) under reflux. The ethanol extract was filtered and concentrated under reduced pressure, and then resuspended in distilled water at the concentration of 100 mg/mL. Thereafter, the extracts were subjected to D101 macroporous resin, and eluted using water and 70% ethanol. The total glycosides were eluted in 70% ethanol, whereas the ethanol elution was collected, concentrated *in vacuo* and freeze-dried. The fingerprint of this extract using an ultra-performance liquid chromatography (UPLC)-photodiode array (PDA) was performed; the result is shown in Figure 1. Four main components of total glycosides from *P. hookeri* were identified, included sweroside (peak 1), loganin (peak 2), sylvestroside I (peak 3) and cantleyoside (peak 4). The extract was suspended in 0.5% sodium carboxymethyl cellulose (CMC) before administration to animals during the experiment.

### Chemicals

Complete Freund’s adjuvant and carrageenan were provided by Sigma Chemical Co. Ltd. (St. Louis, MO). Lyophilized Bacillus Calmette–Guérin vaccine was supplied by Beijing Institute of Biological Products (Beijing, China). Assay kits of SOD, MDA and NO were purchased from Jiancheng Bioengineering Institute (Nanjing, China). NF-κB p65 mouse monoclonal antibody was supplied by Santa Cruz Biotechnology (Santa Cruz, CA). Horseradish peroxidase (HRP)-labelled anti-mouse IgG and DAB kit were from Beijing Zhongshan Golden Bridge Biotechnology Co., Ltd. (Beijing, China). All other chemicals were of analytical grade.

### Animals

Male Sprague–Dawley (SD) rats (220–240 g) as well as female and male Kunming (KM) mice (18–22 g) were obtained from the Institute of Laboratory Animal, Sichuan Academy of Medical Scientific & Sichuan Provincial People’s Hospital (Chengdu, China). The animals were allowed to acclimatize to the laboratory environment for 7 days prior to the experiment. They were kept in plastic cages at 24 ± 2 °C with free access to pellet food and water and on a 12 h light/dark cycle. Animal welfare and experimental procedures were strictly adhered to, in accordance with the Guide for the Care and Use of Laboratory Animals published by the US National Institutes of Health (NIH Publication No. 85-23, revised 1996).

### Hot plate test

This test was performed according to the method previously described (Li et al. 2011). Mice were placed on a hot plate maintained at 55 ± 0.5 °C. The time for paw licking or jumping was taken as the latency time. And, the mice with latency time greater than 30 s or less than 5 s were eliminated. The mice were tested twice prior to drug administration and the mean latency time of each mouse was considered as their basic pain threshold. The latency time was then determined at 15, 30, 60 and 120 min after the administration of drug. A single intraperitoneal injection of morphine was considered as the positive control (Li et al. 2011).

### Acetic acid-induced abdominal writhing reflex and peritoneal permeability

The method was used according to the method previously described by Zhang et al. (2009). The control mice received vehicle (0.5% CMC, 10 mL/kg, i.g.) and positive group received indomethacin (25 mg/kg, i.g.), total glycosides of *P. hookeri* at doses of 28, 56 and 112 mg/kg were orally administered to each mouse daily for 5 days. One hour after treatment, 0.7% acetic acid solution (10 mL/kg) in normal saline was injected intraperitoneally to mice. Then, the incubation period of writhing reflex was observed and the number of abdominal writhing response in a period of 0–15 min was immediately counted.

In the peritoneal permeability test, 1 h after drug treatment, the animals received an intravenous injection of Evans blue dye (10 mg/kg), followed by an intraperitoneal injection of 0.7% acetic acid. Twenty minutes after the injection of acetic acid, the mice were killed by cervical dislocation. Peritoneal fluid was collected by rinsing the cavity with 5 mL of normal saline, followed by centrifugation at 3000 rpm for 5 min. The absorbance of the supernatant was measured at 595 nm using a spectrophotometer.

### Xylene-induced ear oedema

The xylene-induced ear oedema test was previously described (Zhang et al. 2009). The control mice received vehicle (0.5% CMC, 10 mL/kg, i.g.) and positive group received indomethacin (25 mg/kg, i.g.), total glycosides of *P. hookeri* at doses of 28, 56 and 112 mg/kg were orally administered to each mouse daily for 5 days. One hour after the last drug administration, a total of 20 μL of xylene was given on the surfaces of the right ear lobe. One hour later, the animals were sacrificed by cervical dislocation, circular sections of the right and left ears were then taken with a cork borer (diameter of 8 mm) and weighed. The left ear was considered as control. Ear oedema was expressed as the difference value of the right and left ears.

### Carrageenan-induced paw oedema

The test of carrageenan-induced rat paw oedema was done using a previously reported technique (Morris 2003). Rats were orally treated with vehicle (0.5% CMC, 10 mL/kg) and indomethacin (12.5 mg/kg), as well as total glycosides of *P. hookeri* (14, 28 and 56 mg/kg) for 7 days. After 1 h of the last treatment, rats were intradermally injected with 1% w/v carrageenan into the sub-plantar tissue of the right hind paw. The contra-lateral hind paws were intradermally injected with 0.1 mL of normal saline as control. Paw volume was measured by a plethysmometer at 0, 1, 2, 3 and 4 h after carrageenan injection.

### Agar-induced granuloma formation

The test for agar-induced granuloma formation was described in a previous study (Endo et al. 1981). The test samples, indomethacin and vehicle were administered orally once daily for 7 days. At the first day of treatment, 0.4 mL of sterilized 2% agar solution was subcutaneously injected into the back of each mouse. Thereafter, 24 h after the last treatment, animals were sacrificed by cervical dislocation. The agar, which was surrounded by granuloma tissue, was removed and weighed.

### Adjuvant-induced arthritis (AA) induction and drug treatment

Arthritis was induced by a single intradermal injection of 100 μL of complete Freund’s adjuvant containing 10 mg/mL of lyophilized *Mycobacterium tuberculosis* into hind paw of SD rats (Pearson 1963). In the control group, the same volume of liquid paraffin alone was given. AA induction day was designed as day 0. The animals were randomly divided into six groups of 10 animals each as follows: Group I: Normal control rats + vehicle; Group II: AA contral rats + vehicle; Group III–V: AA rats + total glycosides of *P. hookeri* (14, 28 and 56 mg/kg, respectively); Group VI: AA rats + nimesulide (33.33 mg/kg, as the positive control). Rats were pretreated with drugs or vehicle for 3 days before AA induction, and then continuously administered for 30 days after AA induction.

### Evaluation of paw swelling, pain threshold and arthritic score in AA rats

The primary paw swelling (volume, mL) was measured via the water displacement method with a plethysmometer at 6, 12, 24, 36, 48 and 72 h after adjuvant injection. The secondary paw swelling (volume, mL) was determined via the water displacement method at 12, 18, 24, 30 days after AA induction. The pain threshold (pressure pain, g) of injected hind paws were evaluated by an algometer (YLS-3E, Jinan Yiyan Technology Development Co., Ltd., Jinan, China) at 7, 14, 21, 30 days after AA induction (Ahmed et al. 2012). The measurement of the arthritis score was performed at 12, 18, 24, 30 days after AA induction based on the following criteria (Esser et al. 1995): 0 = normal; 1 = mild, slight swelling and erythema limited to individual joints; 2 = moderate, moderate swelling and erythema of joints; 3 = severe redness and swelling of ankle; 4 = severe redness and swelling of the whole paw with severely deformed joints. The maximum score for each rat was 12.

### Biochemical assays in AA rats

On the 30th day, rats were anesthetized by 10% chloral hydrate (10 mL/kg). The blood samples without anticoagulants were collected from femoral artery, then, centrifuged at 3600 rpm for 10 min, and the supernatants were collected. The serum levels of SOD, MDA and NO were measured by the commercial diagnostic kits using chemical colorimetric method.

### Histopathology

The synovial tissues were isolated after the animals were sacrificed, and then fixed in 10% buffered formalin for 24 h. Tissues were dehydrated, processed, embedded in paraffin, sectioned and stained with haematoxylin and eosin. The sections were examined under a light microscope and photographs were taken.

### Immunohistochemistry

For immunohistological staining of NF-κB p65, paraffin sections of synovial tissue were dewaxed with xylene, and hydrated with gradient ethanol. Then, sections were treated in a microwave oven at low power for 10 min in 0.01 M sodium citrate buffer (pH = 6.0) and blocked with 10% goat serum for 1 h at room temperature. Next, the sections were stained with anti-NF-κB p65 (1:100) mouse monoclonal antibody at 4 °C overnight. Sections were then washed with TBST, and endogenous peroxidase was inactivated using 3% hydrogen peroxide for 20 min. Subsequently, sections were incubated with the HRP-labelled secondary antibody (1:1000) for 1 h at room temperature, and antibody-binding sites were visualized by DAB kit for 15 min. Thereafter, sections were stained with haematine for 10 min, dehydrated and cleared by gradient ethanol and xylene, respectively. Finally, the samples were observed under a light microscope after sealing with neutral balsam on slides. The expression of NF-κB p65 was represented as integrated optical density (IOD).

### Statistical analyses

Data were expressed as mean values ± standard deviation. All data analysis was tested by one-way analysis of variance for multiple comparisons with LSD-test. The statistical differences were considered to be significant at the *p* < 0.05 level.

## Results

### Effects of total glycosides from *P. hookeri* on several hyperalgesia animal models

**Figure 2.**
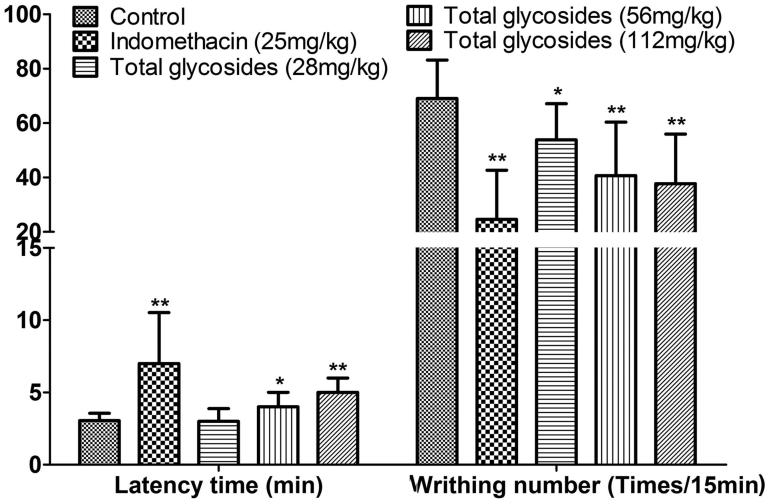
Effect of total glycosides from *P. hookeri* on acetic acid-induced abdominal writhing in mice. Total glycosides (28, 56 and 112 mg/kg) or indomethacin (25 mg/kg) or vehicle was administered 30 min prior to acetic acid injection. The latency time (A) and reduced number of writhing (B) were recorded for 15 min. All data are represented as mean ± SD, *n* = 10, *p < 0.05, **p < 0.01 vs control.

**Figure 3.**
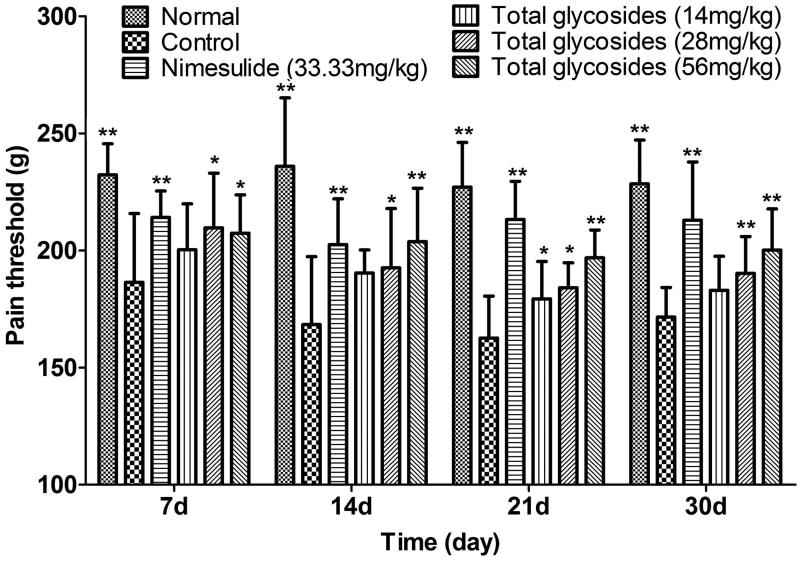
Effect of total glycosides from *P. hookeri* on pain threshold in adjuvant-induced arthritis rats. Arthritis was induced by intradermal injection of 0.1 mL of complete Freund’s adjuvant (CFA). Total glycosides (14, 28 and 56 mg/kg) or nimesulide (33.33 mg/kg) or vehicle was administered 1 h after CFA injection and their daily treatment continued until 30 days after CFA challenge. The pain threshold (pressure pain, g) was assessed on 7, 14, 21 and 30 days after CFA injection via algesimeter. All data are represented as mean ± SD, *n* = 10, *p < 0.05, **p < 0.01 vs control.

**Figure 4.**
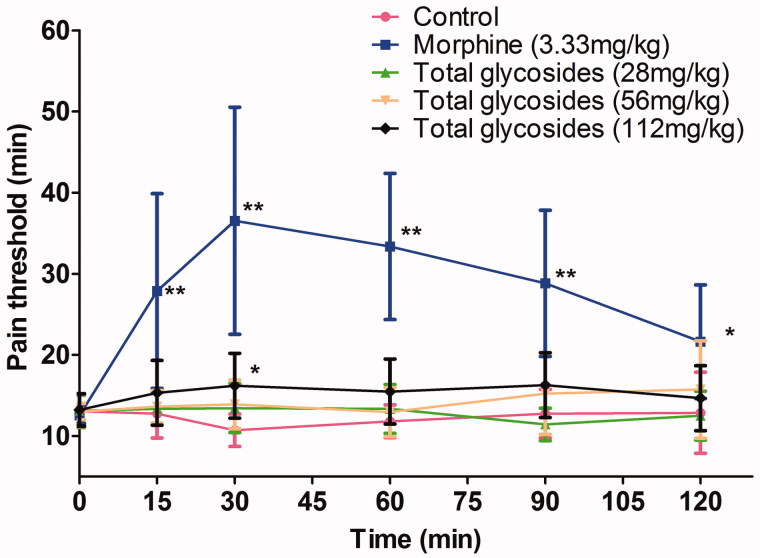
Effect of total glycosides from *P. hookeri* on hot-plate test in mice. Mice were pretreated with total glycosides or morphine (3.33 mg/kg) or vehicle for 3 days. Mice were subjected to the hot-plate test, and the latency time was then determined at 15, 30, 60, 90 and 120 min after administration of drug. All data are represented as mean ± SD, *n* = 10, *p < 0.05, **p < 0.01 vs control.

Firstly, the analgesic effect of total glycosides from *P. hookeri* was assessed in several hyperalgesia animal models. As shown in Figure 2, the total glycosides (56 and 112 mg/kg) exhibited a significant analgesic activity against acetic acid-induced pain by showing the prolonged latency time (4.72 ± 1.18 and 5.03 ± 1.19 vs 3.05 ± 0.51 min, *p* < 0.05–0.01) and reduced number of writhing (40.70 ± 19.60 and 37.70 ± 18.25 vs 69.00 ± 14.17 times, *p* < 0.05–0.01). The analgesic effect of total glycosides was also measured by algometer in AA rats. Compared with the normal rats, AA rats undergoing mechanical stimuli showed a hyperalgesia symptoms characterized by a decrease in pain threshold up to 186.45 ± 29.34 and 162.60 ± 17.97 g after 7 and 30 day of CAF injection, respectively (Figure 3). Administration of total glycosides from *P. hookeri* significantly enhanced the pain threshold of AA rats by up to 21.11% (Figure 3, *p* < 0.05–0.01). However, only administration of total glycosides (112 mg/kg) increased the pain threshold at 30 min after treatment in hot-plate test (Figure 4, *p* < 0.05). These results were comparable to the effects produced by the standard peripheral analgesic drug indometacin (25 mg/kg) and nimesulide (33.33 mg/kg) as well as central analgesic drug morphine (3.33 mg/kg). Taken together, total glycosides from *P. hookeri* possessed analgesic effect on inflammatory pain, but had no obvious effect on the pain caused by thermal stimuli.

### Effects of total glycosides from *P. hookeri* on animal inflammation models

**Figure 5.**
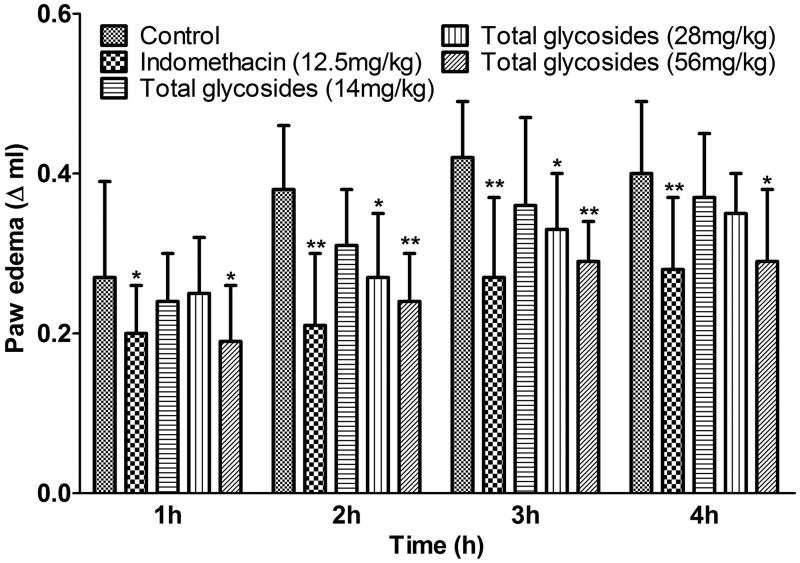
Effect of total glycosides from *P. hookeri* on carrageenan-induced acute paw swelling in rats. Total glycosides (14, 28 and 56 mg/kg) or indomethacin (12.5 mg/kg) or vehicle was administered for 7 days. One hour after last administration, rats were injected with 1% carrageenin into the right hind paw, paw volume was then measured at 1, 2, 3 and 4 h after carrageenin injection. All data are represented as mean ± SD, *n* = 10, *p < 0.05, **p < 0.01 vs control.

**Figure 6.**
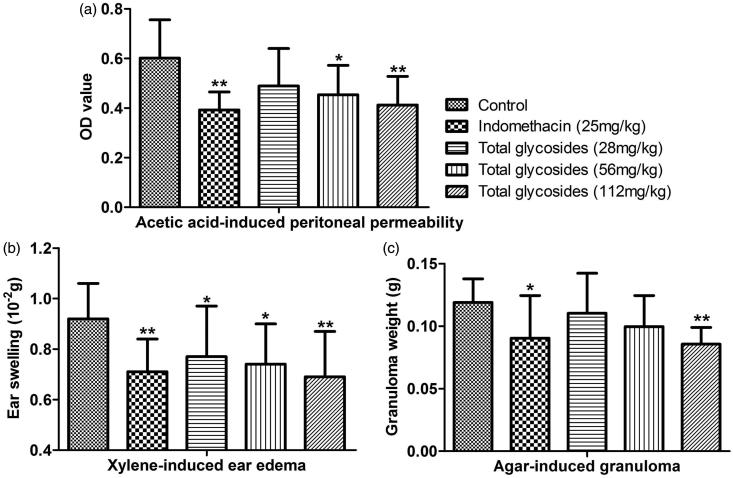
The anti-inflammatory effect of total glycosides from *P. hookeri* on several acute and chronic inflammation models. (a) Xylene-induced ear oedema, (b) acetic acid-induced peritoneal capillary permeability and (c) agar-induced granuloma in mice. All data are represented as mean ± SD, *n* = 10, *p < 0.05, **p < 0.01 vs control.

As shown in Figure 5, the total glycosides from *P. hookeri* (56 mg/kg) inhibited the paw oedema by 29.56, 36.39, 30.64 and 27.00% at 1, 2, 3 and 4 h after carrageenan injection (*p* < 0.05–0.01) respectively, which is comparable with the effects of indomethacin (25 mg/kg) (*p* < 0.05–0.01). Then, after three days treatment of total glycosides from *P. hookeri* (56 and 112 mg/kg) decreased acetic acid-induced peritoneal capillary permeability in mice by about 25% and 32% when compared with vehicle control (Figure 6(a), *p* < 0.05–0.01). Moreover, pretreatment with total glycosides from *P. hookeri* (56 and 112 mg/kg) caused about 20% and 25% suppression in xylene-induced ear oedema in mice, respectively (Figure 6(b), *p* < 0.05–0.01). Indomethacin (25 mg/kg) also exerted significant inhibitory action. Finally, inhibition of the total glycosides from *P. hookeri* (112 mg/kg) on agar-induced granuloma formation (*p* < 0.01) was observed in mice with the inhibition percentage similar as indomethacin (25 mg/kg) (Figure 6(c)). These findings indicated that the total glycosides from *P. hookeri* possessed anti-inflammatory property.

### Effects of total glycosides from *P. hookeri* on paw swelling in AA rats

**Figure 7.**
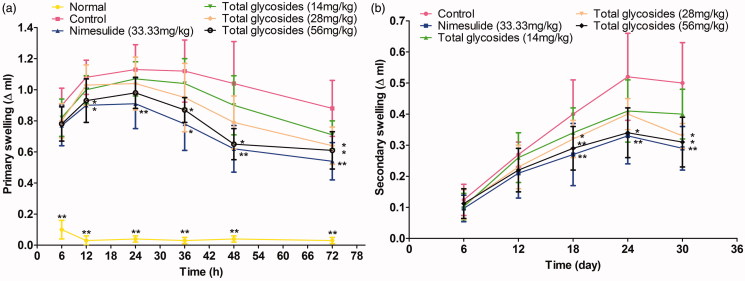
Effect of total glycosides from *P. hookeri* on paw swelling in AA rats. The left hind paw of rats was injected intradermally with 0.1 ml of CFA. Total glycosides (14, 28, 56 mg/kg), or nimesulide (33.33 mg/kg) or vehicle was pretreated for 3 days before CFA injection and then administered 1 h after CFA injection and their daily treatment continued until 30 days after CFA challenge. The primary swelling was measured at 6, 12, 24 36, 48 and 72 h after CFA injection. And secondary paw swelling was assessed on 6, 12, 18, 24 and 30 days after CFA injection. All data are represented as mean ± SD, *n* = 10, *p < 0.05, **p < 0.01 vs control.

To test the anti-arthritic effect of total glycosides from *P. hookeri*, arthritis rats were established by complete Freund’s adjuvant (CFA). After the CFA injection, primary swelling in the left hind paws was induced. Total glycosides (56 mg/kg) decreased the primary swelling by 22.70, 37.50 and 30.57% after 36, 48 and 72 h of CFA injection, respectively (Figure 7(a), *p* < 0.05–0.01). Meanwhile, the secondary arthritis in the right (non-injected) hind paws occurred on day 12 after the CFA injection and the paw swelling reached its peak on day 24 (Figure 7(b)) indicating the success of the model construction. Administration of the total glycosides (56 mg/kg) inhibited the development of secondary paw swelling by 27.50, 34.62 and 38.00% at 18, 24 and 30 days after CFA injection, respectively (Figure 7(b), *p* < 0.05–0.01). Nimesulide (33.33 mg/kg) produced potent inhibition on both primary and secondary paw swelling (Figure 7(a,b), *p* < 0.05–0.01). These results indicated an anti-arthritic effect of the total glycosides from *P. hookeri* on AA rats.

### Effects of total glycosides from *P. hookeri* on arthritis score in AA rats

**Figure 8.**
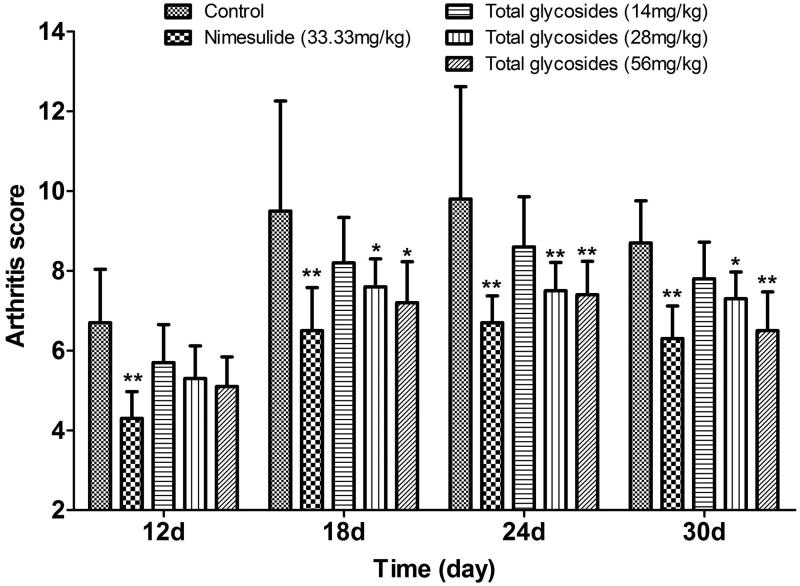
Effect of total glycosides from *P. hookeri* on arthritis score in AA rats. Arthritis score was evaluated on 12, 18, 24 and 30 days after CFA injection. All data are represented as mean ± SD, *n* = 10, *p < 0.05, **p < 0.01 vs control.

In AA rats, systematic secondary response occurred on 12 days after CFA injection, which manifested as a progressively increased arthritis score. Figure 8 demonstrated the effect of total glycosides from *P. hookeri* on arthritis score in AA rats. Treatment with total glycosides from *P. hookeri* (56 mg/kg) diminished the arthritis score by 24.21, 24.49 and 25.29% after 18, 24 and 30 days of CFA challenge (*p* < 0.05–0.01), and the inhibition rates of 28 mg/kg total glycosides were 20.00, 23.47 and 16.09% (*p* < 0.05–0.01), respectively. The resulting efficacy of total glycosides from *P. hookeri* at 56 mg/kg on arthritis was similar to that of nimesulide (33.33 mg/kg).

### Effects of total glycosides from *P. hookeri* on NF-κB p65 expression in AA rats

**Figure 9.**
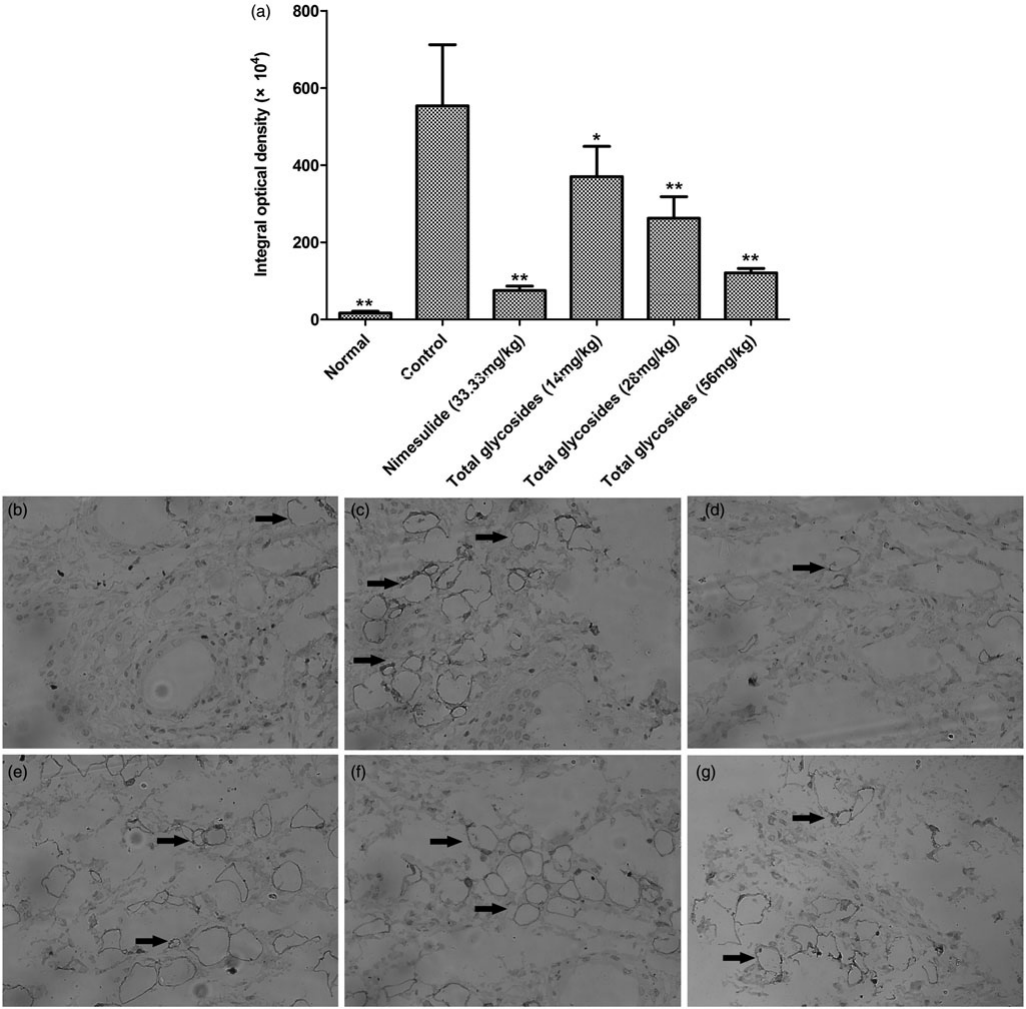
Effect of total glycosides from *P. hookeri* on the expression of NF-κB p65 in synovium of AA rats. The expression of NF-κB p65 in synovial tissues were measured by immunohistochemistry. (a) Quantitative results of NF-κB p65 expression are depicted as integral optical density. All data are represented as mean ± SD, *n* = 6, *p < 0.05, **p < 0.01 vs control. (b–g) Typical images of NF-κB p65 expression in synovial tissues were obtained by the microscope (400× ), (b) normal, (c) control, (d) nimesulide (33.33 mg/kg), (e) 14 mg/kg of total glycosides, (f) 28 mg/kg of total glycosides, (g) 56 mg/kg of total glycosides. Arrows indicate the positive expression.

NF-κB is a crucial transcription factor in various inflammatory responses, including rheumatoid arthritis. To determine whether the anti-arthritic effect of total glycosides from *P. hookeri* was due to alternation of NF-κB, we examined the expression level of NF-κB p65, a key subunit of NF-κB. As shown in Figure 9, elevated expressions of NF-κB p65 were observed in synovial tissues of AA rats compared with the normal control. NF-κB p65 expression was significantly reduced by treatment with total glycosides from *P. hookeri* (14, 28 and 56 mg/kg, inhibition rate were 33.10, 52.64 and 78.16%, respectively, *p* < 0.05–0.01), as well as by nimesulide. This result indicated that total glycosides from *P. hookeri* exerted an anti-arthritic effect, in part, by the inhibition of NF-κB p65 expression.

### Effects of total glycosides from *P. hookeri* on oxidative stress in AA rats

**Figure 10.**
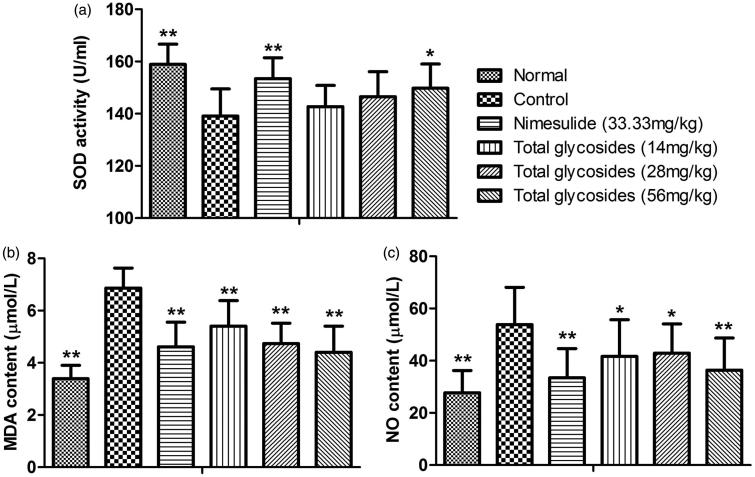
Antioxidant effects of total glycosides from *P. hookeri* in AA rats. (a) Activity of serum SOD and (b) serum levels of MDA and (c) NO are depicted. All data are represented as mean ± SD, *n* = 10, *p < 0.05, **p < 0.01 vs control.

Excessive oxygen free radicals produced by macrophages have a close relationship with joint injury. Therefore, we examined whether the total glycosides from *P. hookeri* altered the oxidative stress in AA rats. Figure 10 showed that the serum levels of MDA (3.39 ± 0.51 vs 6.86 ± 0.77 μmol/L, *p* < 0.01) and NO (27.69 ± 8.56 vs 53.82 ± 14.28 μmol/L, *p* < 0.01) were both increased significantly in AA rats, whereas the serum activity of SOD (158.89 ± 7.76 vs 139.11 ± 10.38 U/mL, *p* < 0.01) was found to be decreased in AA rats. Total glycosides from *P. hookeri* (14, 28 and 56 mg/kg) significantly reversed the elevated serum levels of MDA (Figure 10(b), 5.40 ± 0.98, 4.74 ± 0.78 and 4.40 ± 1.00 μmol/L, respectively, *p* < 0.01) and NO (Figure 10(c), 41.61 ± 14.11, 42.88 ± 11.20 and 36.37 ± 12.34 μmol/L, respectively, *p* < 0.05–0.01), whereas the modest increase of SOD activity (7.66%) in serum was detected only when 56 mg/kg of total glycosides from *P. hookeri* was applied (Figure 10(a), *p* < 0.05). However, nimesulide (33.33 mg/kg) significantly reversed the above abnormal changes (*p* < 0.01). This result showed an important repressor effect of total glycosides from *P. hookeri* on oxidative stress in AA rats, which may contribute to its anti-arthritic effect.

### Effect of total glycosides from *P. hookeri* on synovial histopathology in AA rats

**Figure 11.**
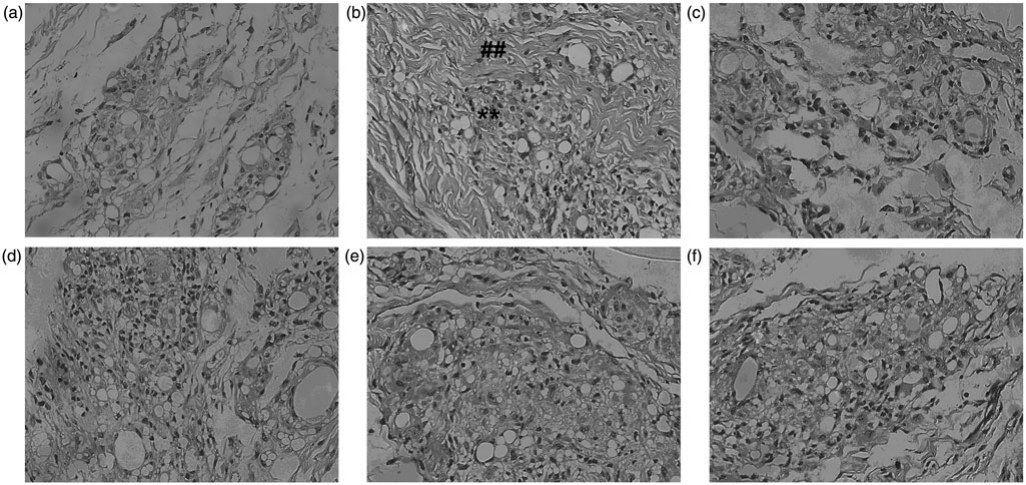
Effect of total glycosides from *P. hookeri* on histopathology of synovium in AA rats. (a) Normal synovial tissue, (b) synovial tissue of AA rats treated with vehicle showed obvious inflammatory cell infiltration (**) and synovial hyperplasia (##), (c) synovial tissue of AA rats treated with nimesulide (33.33 mg/kg), (d–f) synovial tissue of AA rats treated with 14, 28 and 56 mg/kg of total glycosides from *P. hookeri*, respectively. Panels c, d, e and f show reduction in inflammatory cell infiltration and synovial hyperplasia (original magnification  400×).

Histologic evaluation of the synovial tissues in the vehicle-treated animals revealed obvious signs of arthritis, with marked inflammatory cell infiltration and synovial hyperplasia (Figure 11(b)), compared with the normal rats (Figure 11(a)). The degree of inflammatory cell infiltration and synovial hyperplasia was significantly reduced after receiving total glycosides (14, 28 and 56 mg/kg) or nimesulide (33.33 mg/kg).

## Discussion

Natural products are historically invaluable as a source of therapeutic agents. Recently, increasing attentions have been paid on the development of new therapeutic agents from natural products or traditional medicinal plants due to their wider safety range. *P. hookeri*, a famous Tibetan herbal medicine, has been found to have analgesic, anti-inflammatory and anti-cancer effects (Guo et al. 2015; Zhang et al. 2009); however, its effect on RA is seldom reported, and most of the previous studies only focus on the crude extract of *P. hookeri*. RA is a complicated autoimmune joint disease with persistent inflammation and destruction of cartilage and bone as well as chronic hyperalgesia (McInnes & Schett 2011). Recently, the goal of therapy for RA is inhibition of inflammation, prevention of long-term joint damage and attenuation of pain (McInnes & Schett 2011; Singh et al. 2012). Therefore, we investigated the pharmacological effects of total glycosides from *P. hookeri*, the major active components of *P. hookeri*, on RA by assessing its anti-inflammatory and analgesic activities in several animal models, including adjuvant-induced arthritis and carrageenan-induced paw oedema, as well as some models of mechanical, chemical and thermal nociception.

Adjuvant-induced arthritis (AA) in rat is a well-established animal model that shares some features with human RA, including joint swelling, pain, cartilage degradation and loss of joint function (Asquith et al. 2009). One of the most important features of AA is chronic synovitis, including inflammatory cell infiltration and synovial hyperplasia (Ashraf et al. 2010). Hence, AA rats have been used frequently to estimate possible therapeutic agents in the treatment of RA (Asquith et al. 2009). In this study, paw swelling and arthritic scores are indices of measuring the anti-arthritic activity of various agents and were employed to determine the activity of total glycosides from *P. hookeri*. We found that the total glycosides from *P. hookeri*, at the dosages of 28 and 56 mg/kg, significantly decreased the primary paw swelling, as well as the secondary paw swelling and the arthritis score in the later stage of AA. Moreover, histopathological examination further demonstrated the beneficial function of total glycosides from *P. hookeri* in attenuating the major pathological characteristics of RA, such as inflammatory cell infiltration and synovial hyperplasia.

In addition, xylene-induced ear oedema, acetic acid-induced peritoneal permeability and carrageenan-induced paw oedema were performed to further explore the anti-inflammatory activity of total glycosides from *P. hookeri*. These three models are mainly associated with the release of pro-inflammatory mediators, such as substance P, prostaglandins, histamine, etc. (Deraedt et al. 1980; Nantel et al. 1999). In our study, the total glycosides of *P. hookeri* showed a remarkable suppression on inflammation in these models, which may be related to the inhibition of inflammatory mediators. Furthermore, the total glycosides of *P. hookeri* (56 mg/kg) also decreased agar-induced granuloma formation, a sub-chronic inflammatory model (Endo et al. 1981). Our results were consistent with the previous finding reported by Zhang et al. (2009). Meanwhile, we also have identified four main compounds in the total glycosides from *P. hookeri*, including sweroside, loganin, sylvestroside I and cantleyoside (Figure 1). Ryu et al. (2010) reported sweroside and loganin at oral doses of 0.1 and 1 mg/kg showed significant anti-inflammatory and analgesic effects on croton oil-induced ear swelling and acetic acid-induced writhing responses, which may contribute to the anti-inflammatory potency of the total glycosides from *P. hookeri*. Taken together, these changes suggested that the total glycosides from *P. hookeri* possessed anti-inflammatory and anti-arthritic effects.

It is well-known that the pleiotropic transcription factor NF-κB plays a crucial role in the pathogenesis of RA by up-regulating the expression of multiple genes, such as pro-inflammatory cytokines and chemokines (Simmonds & Foxwell 2008). And the products of these genes, in turn, coordinately enhance inflammatory reactions resulting in further activation of NF-κB (Hayden & Ghosh 2011). Indeed, persistent overexpression or overactivation of NF-κB has been observed in the synovial tissues of RA patients and arthritic animals compared with those of normal controls (Handel et al. 1995; Yang et al. 2010). Interestingly, the most prevalent activated form of NF-κB is the heterodimer p65 and p50, which are abundant in RA (Simmonds & Foxwell 2008). Therefore, NF-κB is essential for inflammation triggering and amplifying during RA, which also has been considered as an attractive drug target molecule for RA therapy (Roman-Blas & Jimenez 2006). NSAIDs, some DMARDs, glucocorticoids and some natural products have been described to decrease NF-κB activation (Simmonds & Foxwell 2008). In the present study, the total glycosides of *P. hookeri* treatment could significantly decrease the expression of NF-κB p65 in the synovial tissues in affected joints as determined by immunohistochemistry method, indicating that total glycosides of *P. hookeri* exerted anti-inflammatory effect possibly correlated with inhibiting the NF-κB signalling pathway. In addition, Wu et al. reported an inhibitory effect of some iridoids isolated from *P. hookeri* on TNF-α-induced NF-κB-dependent promoter activity (Wu et al. 2014a), which further confirmed the results obtained in the present study.

Reactive oxygen species (ROS), continuously produced inside the cell following exposure to various exogenous and endogenous stimulus, also can be neutralized by the endogenous antioxidants, which creates a balance between the ROS generation and the antioxidants (Sun 1990). An imbalance between the generation and inactivation of ROS results in severe oxidative stress, cellular function irregularities and different pathological conditions including RA (Kundu et al. 2012). RA is always accompanied with increased lipid peroxidation and decreased antioxidants in human and animal models (Kundu et al. 2012; Shanmugarajan et al. 2009). In this study, a significant elevation of two oxidation products, MDA and NO, as well as decreased the serum activity of SOD antioxidant were observed after CFA challenge. Total glycosides of *P. hookeri* triggered a significant protective effect by reducing the contents of MDA and NO, and increasing the activity of SOD in AA rats. Furthermore, the transcription of NF-κB-dependent genes influences the levels of ROS in the cell, and in turn, the transcription of NF-κB is also regulated by the levels of ROS (Morgan & Liu 2011). Hence, we speculated that the antioxidation effect of total glycosides of *P. hookeri* is due, at least in part, to its inhibitory effect on the expression of NF-κB p65. Based on our findings, the antioxidation effect of total glycosides of *P. hookeri* may also contribute to its regulation on NF-κB signalling.

Chronic pain is another important clinical feature of RA, which greatly diminishes quality of life (Smolen et al. 2016). Therefore, we performed a series of experiments to explore the analgesic activity of the total glycosides from *P. hookeri* in several mechanical, chemical and thermal nociception of rodent models. AA rats are a well-characterized model of persistent pain hypersensitivity, acetic acid-induced writhing in mice is another classic model of acute pain (Hegen et al. 2008). The release of several pro-inflammatory cytokines and mediators including TNF-α and PGE_2_ contribute to the nociceptor sensitization and a decrease in nociceptive threshold in the two pain models (Deraedt et al. 1980; Lima-Garcia et al. 2011). Herein, total glycosides from *P. hookeri* caused a significant suppression in the mechanical and chemical hyperalgesia induced by CFA and acetic acid, respectively. However, total glycosides from *P. hookeri* failed to attenuate the thermal hyperalgesia in hot plate test. These results suggested an inhibitory effect of the total glycosides from *P. hookeri* on peripheral inflammatory pain, while central nervous system is not involved in its action. Meanwhile, a number of recent studies indicate that NF-κB is implicated in the pathogenesis of inflammatory hyperalgesia (Niederberger & Geisslinger 2008). Hence, these results, in conjunction with the significant inhibition of inflammation and oedema, imply that the anti-arthritic, analgesic effect of total glycosides from *P. hookeri* may, in part, result from its regulation of NF-κB p65 expression.

## Conclusions

In summary, the total glycosides from *P. hookeri* possess potent anti-arthritic and analgesic activity in several animal models. All these actions may be attributed to its ability in inhibiting the expression of NF-κB p65 as well as its antioxidant activity. However, the precise mechanisms of action and the active fractions among total glycosides from *P. hookeri* remain to be further investigated.
